# Subcortical Brain Regions Associated With Seizure Risk in Patients With IDH Mutated Diffuse Gliomas

**DOI:** 10.1002/brb3.70477

**Published:** 2025-04-09

**Authors:** Ann Westermark, Markus Fahlström, Sadia Mirza, Maria Zetterling, Eva Kumlien, Francesco Latini

**Affiliations:** ^1^ Department of Medical Sciences, Neurology Uppsala University Uppsala Sweden; ^2^ Department of Surgical Sciences, Molecular Imaging and Medical Physics Uppsala University Uppsala Sweden; ^3^ Department of Medical Sciences, Neurosurgery Uppsala University Uppsala Sweden

**Keywords:** connectivity, diffuse glioma, seizures, white matter tracts

## Abstract

**Intro:**

Seizure incidence in diffuse glioma ranges between 60% and 90%. This study aimed to investigate the association between seizures and diffuse glioma in subcortical and cortical brain regions, including white matter tracts.

**Methods:**

Adult patients with diffuse glioma at Uppsala University Hospital from 2005 to 2021 were analysed retrospectively. The relationship between tumour location in specific brain voxels and preoperative seizures was examined concerning white matter tract involvement. Tumour volumes were segmented based on T2‐weighted or FLAIR MRI after spatial normalisation to standard space (MNI) and combined to create a location‐specific frequency map.

**Results:**

Of the 93 patients meeting the inclusion criteria, 70 (75%) experienced seizures. A significant decreased risk was found in tumours present within the left fronto‐mesial and dorsal voxel (A3C1S1). Increased seizure risk was found in tumours located in the left supramarginal and posterior insular voxel (A4C2S3). The voxels differed in terms of type and extent of white matter networks. Additionally, there was a difference in seizure risk and voxel associations between oligodendrogliomas and astrocytoma, with specific voxels associated with seizures identified in both groups.

**Conclusion:**

The study provides new insights into the epileptogenic potential of diffuse gliomas in relation to their spatial distribution, highlighting the need to analyse both cortical and subcortical localisation of tumours. The observed differences in seizure risks across brain regions underscore the need for personalised post‐surgery treatment strategies and further research to understand the pathophysiology of brain tumour‐related epilepsy, BTRE.

## Graphical abstract

Seizures occur in 75% of patients with diffuse glioma, with risk varying by tumour location. This study maps seizure‐associated brain regions, highlighting differences between glioma subtypes and the role of white matter tracts. Findings support personalised treatment strategies and deeper exploration of brain tumour‐related epilepsy (BTRE).

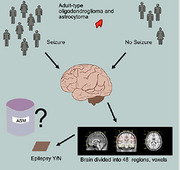

## 1 Introduction

Brain tumour‐related epilepsy (BTRE) is common in patients with diffuse gliomas, with an overall incidence of 60–90%. The mechanism underlying epileptogenesis in brain tumours remains unclear, but a multifactorial cause, depending on brain tumour type and infiltrated area is likely (Armstrong et al. 2016a; Seidel et al. 2022; van Breemen et al. 2007). Epileptogenesis presumably begins in the cortex in close proximity to the diffuse gliomas, which by themselves are usually electrically inert (Duffau et al. 2003; Pallud et al. 2013).

According to the 2021 WHO classification of tumours of the CNS, adult‐type diffuse glioma is classified into three types: astrocytoma isocitrate dehydrogenase (IDH)‐mutant, oligodendroglioma IDH‐mutant and 1p/19q codeleted and glioblastoma IDH‐wild type (Louis et al. 2021). Diffuse gliomas infiltrate extensively but proliferate slowly which provides an opportunity for cortical adaptation mechanisms and enables both functional and morphological reorganisation of the brain (Louis et al. 2021; Smits et al. 2015). Subcortical pathways play an important role in tumour invasiveness and diffuse glioma cells migrate along white matter tracts, with a higher invasion rate than in grey matter (Duffau 2022; Giese et al. 2003; S. Zhang et al. 2023). Typically, neurological deficits are not present at the onset of the disease. It has been suggested that the brain tumours change the neuronal networks leading to loss of function and an increased risk of seizures (Rudà et al. 2012; Smits et al. 2015). Epilepsy is considered a network disease, where focal epilepsy, such as BTRE is believed to originate, spread and terminate in brain networks that also are involved in normal physiological brain function (Berg and Scheffer 2011; Engel et al. 2013; Royer et al. 2022).

The seizure risk varies depending on tumour location, occurring more frequent in frontal, temporal and parietal regions, but less often in occipital and infratentorial regions (Audrey et al. 2024). Seizures are more prevalent in oligodendrogliomas because they more frequently involve the cortex compared to astrocytoma (Chang et al. 2008; Tork and Atkinson 2024). The risk of BTRE, in both astrocytoma and oligodendroglioma, is also affected by age, sex and tumour grade decreasing with age, increasing in males (Armstrong et al. 2016b; Seidel et al. 2022).

Previous studies investigating the relationship between preferential location of diffuse glioma and white matter structures, described astrocytoma as primarily infiltrating white matter tracts related to associative and projective functions, particularly within the left frontal‐temporal‐insular regions. In contrast, oligodendrogliomas tend to invade fibres in the frontal‐mesial regions bilaterally, through more extensive white matter networks that encompass associative, commissural, and projective pathways (Armstrong et al. 2016a; Smits et al. 2015).

The incidence of preoperative seizures in gliomas patients varies depending on tumour location within the cerebrum, which has been a subject of debate. Some argue that gliomas in the insular cortex are more prone to cause seizures, while others believe that gliomas in the temporal lobe are associated with a higher seizure risk. A large review of over 4000 glioma patients revealed that frontal lobe gliomas were linked to a high seizure risk, whereas occipital lobe tumours were associated with a lower risk (Chang et al. 2008; J. Zhang et al. 2019).

Despite the diffuse invasiveness of gliomas throughout white matter, the standard radiological classification of gliomas is based on lobe involvement rather than subcortical and white matter structures or specific portions of the major lobes. Several studies have observed a correlation between changes in white matter tracts and epilepsy in glioma patients. However, the underlying mechanisms and full extent of these changes remain largely unexplored. Previous research has suggested that diffuse glioma BTRE affect the structural integrity of subcortical structures. Despite these findings, the exact mechanisms underlying tumour‐induced seizures remain poorly understood (Bouwen et al. 2018; Weller et al. 2014; S. Zhang et al. 2023).

The aim of this study is to investigate if specific cortical and subcortical regions, including white and grey matter, are linked to higher risk of seizure in patients with diffuse glioma, using a standardised subcortical anatomical and functional reference.

## 2 Methods and Materials

### 2.1 Study Population

From two cohorts, 427 adult patients (≥ 18 years) with first‐time surgery (resection or biopsy) were retrospective collected at the Department of Neurosurgery at Uppsala University Hospital between February 2005 and June 2021. The first cohort consisted of diffuse low‐grade glioma according to prior histological and radiological criteria, recruited between February 2005 and December 2015. The second cohort included all patients who underwent surgery due to radiological suspicion of a brain tumour between August 2014 and June 2021. Both cohorts were scanned at the same site, the Department of Neurosurgery at Uppsala University Hospital, using similar protocols. Minor differences in MRI parameters over the years are acknowledged as a limitation. Seizures were evaluated at the time of tumour detection. Finally, 93 patients with molecularly confirmed diffuse astrocytoma or oligodendroglioma grades 2 and 3 according to the 2021 WHO classification of CNS were included. All other tumours including relapses were excluded. Two patients were excluded due to previously known epilepsy.

**FIGURE 1:**
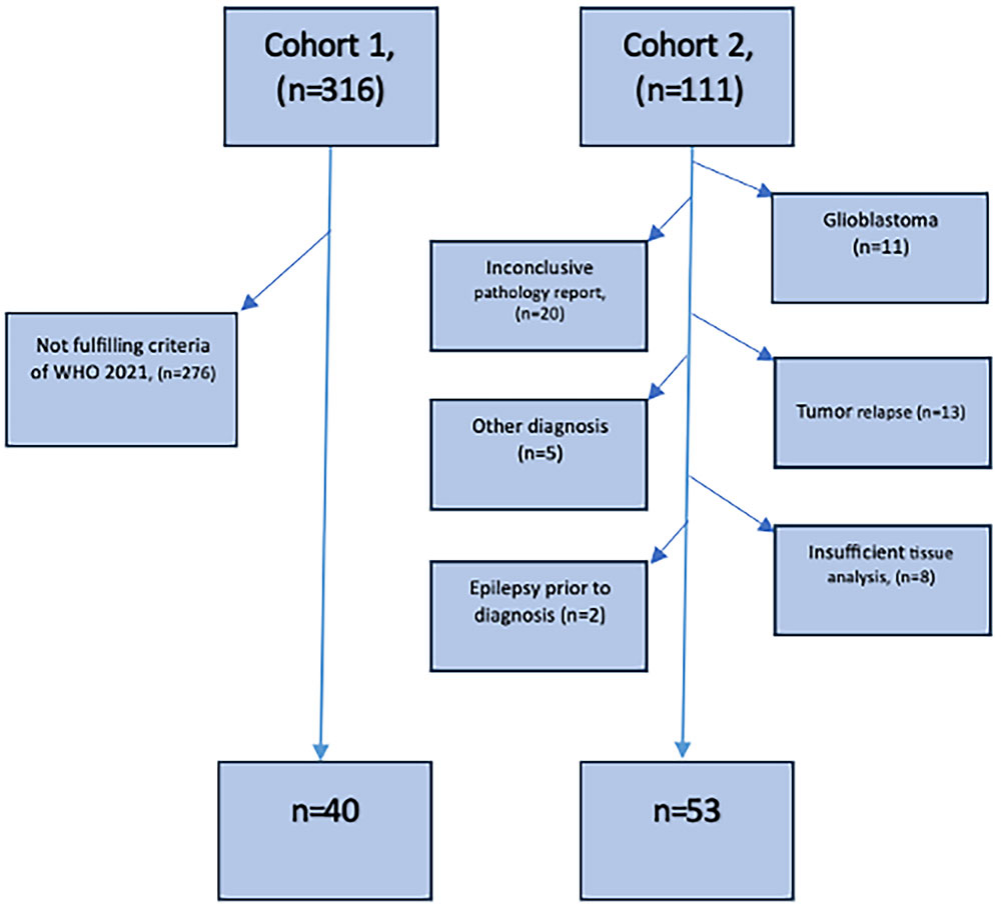
Flowchart detailing participant selection, showing initial screening numbers, exclusion criteria with excluded participant counts, and final inclusion totals for the analysis.

Ninety‐three patients met the inclusion criteria (Figure 1). Medical records with demographics, comorbidity, pathology report, neuroimaging, seizure history, and anti‐seizure medication were collected. Both focal and bilateral seizures were included, and no distinction was made between them regarding seizure occurrence.

### 2.2 Magnetic Resonance Imaging

All patients were scanned on a 3.0 Tesla MRI system (Achieva, Philips Healthcare) using a 32‐channel head coil. In cohort 1 the imaging protocol included an axial 2D T2‐weighed turbo spin echo (T2‐TSE) with repetition time = 3000 ms, echo time = 80 ms, flip angle = 90° and resolution = 0.45 × 0.45 × 5 mm^3^ and an axial 2D T2‐fluid attenuated inversion recovery (FLAIR) image with repetition time = 11,000 ms, echo time = 125 ms, inversion time = 2800 ms, flip angle = 90° and resolution = 0.45×0.45×5 mm^3^. In cohort 2 the imaging protocol included a 3D T2‐FLAIR with repetition time = 4800 ms, echo time = 270 ms, inversion time = 1650 ms, flip angle = 90° and resolution = 1×1×1 mm^3^. In 2015, the MRI system was upgraded to the dStream platform which affects both cohorts. However, no changes to the protocols as given above were done after the upgrade.

### 2.3 Ethics

The study was approved by the regional ethics committee of Uppsala (Dnr 2015/210). The ethics committee waived the need for informed consent for patients operated between 2005 and 2015. Written informed consent was collected from patients operated after January 2015.

### 2.4 Tumour Topography and Seizure Risk

**FIGURE 2:**
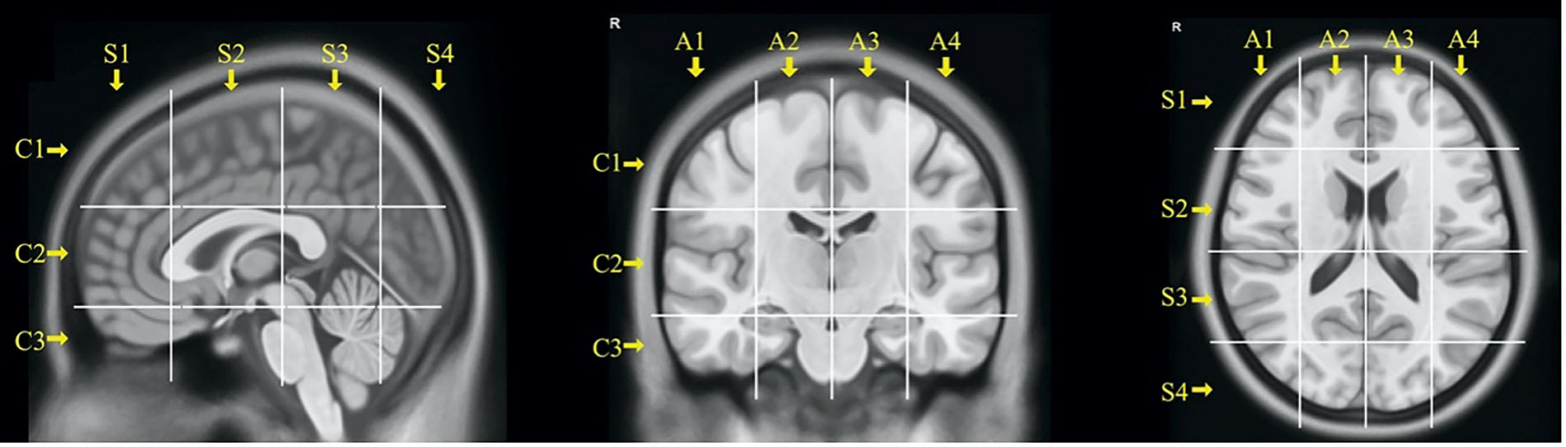
Illustrating MRI images with grey lines representing the 48 voxels of the BG system, along with the axial, coronal, and sagittal positions of each voxel.

To standardise a sublobar analysis of tumour extension, the BGC system was used. This approach allowed for the visualisation of deeper anatomical landmarks and the localisation of white matter structures and tracts within each voxel. Tumour volumes were segmented based on T2‐weighted or FLAIR MRI, normalised into Montreal Neurological Institute (MNI) space. The brain was then divided into 48 voxels named according to the axial (A1–4), coronal (C1–3) and sagittal (S1–4) position, as illustrated in Figure 2. Once the grid was created, high signal on T2 FLAIR or T2 TSE images were used to identify the tumour and describe the cortical subcortical position of the tumour by the type of voxels involved and an indirect measure of tumour invasiveness by counting the number of voxels involved. A white matter tract atlas was generated using MRI with diffusion tensor imaging (DTI) and tractography. Eight major white matter bundles were reconstructed according to the HCP Diffusion MRI Template, as previously described (Latini et al. 2019). The inferior fronto‐occipital fasciculus (IFOF), corticospinal tract (CST), uncinate fasciculus (UF), anterior indirect component of superior longitudinal fasciculus (aSLF) and posterior indirect component (pSLF), arcuate fasciculus (AF), frontal aslant tract (FAT), cingulum (Ci) were reconstructed into MNI following the anatomical criteria already published with the Brain Grid DTI reference atlas (Ius et al. 2011; Latini et al. 2019, 2020).

Segmented tumours in MNI standard space were combined into two different tumour occurrence weighted maps for patients with and without seizures and normalised to the total number of tumours within each group, respectively. The tumour occurrence weighted maps were then projected onto the reconstructed white matter tracts to visually assess tract‐specific differences between patients with and without seizures (Latini et al. 2020).

### 2.5 Statistics

Seizure occurrence was evaluated at tumour detection as a dichotomous variable (yes/no), with logistic regression and odds ratios (OR) identified as the most suitable analytical methods to assess voxel‐specific tumour involvement and its association with seizure risk. Univariate logistic regression was performed for each of the 41 voxels, resulting in 41 individual tests. Variables showing significant or clinically relevant associations in the univariate analysis were subsequently included in a multivariate logistic regression model to adjust for confounders such as sex, tumour volume, and border sharpness, using a stepwise method with conditional entry. Odds ratios with 95% confidence intervals (CIs) quantified the strength of associations, with statistical significance set at *p* < 0.05.

Nominal *p* values were reported without adjustment for multiple comparisons to systematically test the hypothesis and identify voxel‐specific associations with seizure risk. Subgroup analyses were conducted for oligodendrogliomas and astrocytoma (grades 2 and 3) using univariate logistic regression followed by multivariate regression analysis to identify potential differences in risk patterns between tumour subtypes.

Statistical analyses were conducted using IBM SPSS Statistics (version 28.0.1.0). This hypothesis‐driven study aimed to test the predefined association between voxel‐specific tumour involvement and seizure risk.

## 3 Results

### 3.1 Study Population

Of the 93 patients who met the inclusion criteria, 58 (62%) were men. The overall mean age was 42 years (ranging from 20 to 74 years), and 70 patients (75%) experienced seizures. For details, refer to Table 1.

**TABLE 1:** Patient characteristics.

Tumour type and seizure occurrence	*n* (%)	
All tumours, with seizures	70 (75%)	
Astrocytoma, grade 2	34 (37%)	
With seizures	27 (29%)	
Astrocytoma, grade 3	16 (17%)	
With seizures	13 (14%)	
Oligodendroglioma, grade 2	31 (33%)	
With seizures	21 (23%)	
Oligodendroglioma, grade 3	12 (13%)	
With seizures	9 (10%)	
**Sex**		
Males	58 (62%)	
With seizures	49 (53%)	
Females	35 (38%)	
With seizures	21 (23%)	
**Age**	**Years (min–max)**	
Mean	42	
Median	40 (20–74)	
**Volume (mL)**	**Mean (min–max)**	
All tumours	62 (1.4–183.3)	
With seizures	60.2 (3.9–183.0)	
**Border**	***n***	
Diffuse	54 (58%)	
With seizures	42 (45%)	
Voxel amount, average per patient	8.65	
Sharp	29 (31%)	
With seizures	28 (30%)	
Voxel amount, average per patient	5.72	
**Side (*n*)**	***n***	
Left	53 (57%)	
With seizures	39 (42%)	
Right	37 (40%)	
With seizures	29 (31%)	
Bilateral	3 (3%)	
With seizures	2 (2%)	
**Voxels with tumour occurrence (*n*)**	**Median (min–max)**	
All tumours	8 (1–16)	
With seizures	8 (1–14)	
No seizures	7 (2–16)	
Astrocytoma grade 2	4 (2–16)	
With seizures	7 (2–14)	
Astrocytoma grade 3	8 (4–16)	
With seizures	8 (2–12)	
Oligodendroglioma, grade 2	8 (2–16)	
With seizures	5 (1–14)	
Oligodendroglioma, grade 3	8 (2–16)	
With seizures	8 (4–16)	

### 3.2 Tumour Features and Seizure Risk

**FIGURE 3:**
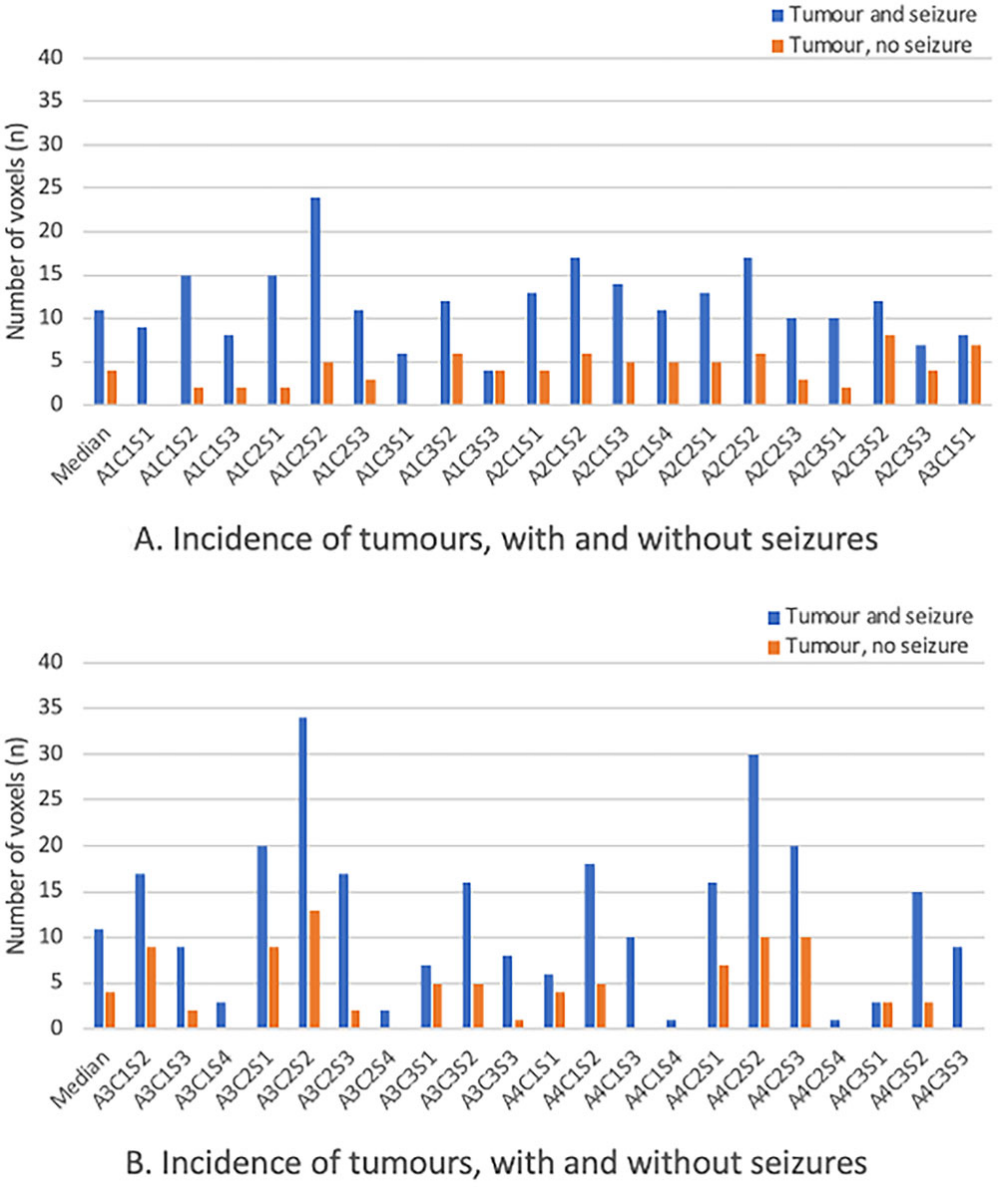
(A, B)Distribution of seizure occurrence and tumour presence. Voxels are coded as A, C, and S to represent their positions: axial (A), coronal (C), and sagittal (S). Voxels A1 (lateral) and A2 (medial) are located in the right hemisphere, while A3 (medial) and A4 (lateral) are located in the left hemisphere. Refer to Figure 2 for MRI images, which illustrate the 48 voxels of the basal ganglia (BG) system, with grey lines indicating the axial, coronal, and sagittal positions of each voxel.

A descriptive analysis to illustrate the distribution between all tumours with or without seizure for each single voxel is displayed in Figure 3A and B. For a detailed description of the contents of each of the 48 voxels, including the white matter, refer to previous publications outlining this technique (Latini et al. 2019, 2020).

First, all variables were analysed using univariate logistic regression (stepwise) (see Table 2).

**TABLE 2:** Univariate analysis for seizure risk.

Seizure risk analysis	OR	95% CI	*p* Value
Male sex	3.6	1.36–9.68	0.01*
Voxel count per tumour	1.0	0.87–1.14	0.93
Diffuse border (diffuse = 1, sharp = 0)	3.5	0.3–1.9	0.51
Volume (cm^2^)	1.0	0.99–1.01	0.60
Age diagnosed	1.0	0.96–1.03	0.62
A4C2S3	8.8	1.1–69.7	0.04*
A3C1S1	0.3	0.1–0.9	0.04*

*Significance (*p* < 0.05; 95% CI). The remaining 39 voxels, which are not statistically significant, have been moved to the Appendix (Table A1) due to space limitations.

The analysis revealed an increased risk of epileptic seizures with tumour presence and a decreased risk with tumour presence in A3C1S. These voxels differ both in anatomical position and white matter connectivity. A4C2S3 is a lateral posterior voxel corresponding to the left cortical areas (A4), situated between the posterior insular cortex and the angular and supramarginal gyrus (C2) and including the temporoparietal junction (S3). The posterior voxel consists of parts of the parietal operculum, the temporoparietal junction, the supramarginal and angular gyrus, extending to the dorsolateral occipital cortex on the lateral surface of the hemisphere. A3C1S1, in contrast, is located anterior dorsally, between superior frontal sulcus and the midline in the axial plane on the left side (A3), frontally toward the superior part of the corpus callosum in the coronal plane (C1) and in the sagittal plane projecting from the anterior cingulum to the dorsal prefrontal cortex as part of the superior frontal gyrus (S1). This includes the supplementary and cingulate eye field area (SCEF), which is a part of medial superior frontal gyrus regions (Seitzman et al. 2019).

The final model from the univariate analysis included two voxels A3C1S1(OR = 6.1, 95% CI: (1.33–28.15), *p* = 0.02) and A4C2S3 (OR = 0.06, 95% CI: (0.01–0.03), *p* = 0.001) and male sex (OR = 7.88, 95% CI: (2.30–27.50), *p* = 0.01). Variables with a significant association in the univariate analysis were subsequently entered into a multivariate logistic regression model (with conditional entry). Due to space limitations, the remaining 39 voxels that did not show statistically significant associations have been moved to the Appendix (Table A1).

Next, seizure risk in oligodendroglioma (grades 2 and 3) and astrocytoma (grades 2 and 3) was analysed separately with univariate analysis and proceeding to multivariate analysis. The results revealed a reduced seizure risk in A1C3S3 (OR 0.05, *p* = 0.0028) in oligodendroglioma. Male sex was related to an increased seizure risk, although this result was not statistically significant (OR 5.3, *p* = 0.1).

In astrocytoma A3C1S1 also showed a decreased seizure risk (OR 0.038, *p* < 0.001), but no correlation between male sex and seizures was found.

Finally, all tumour types and grades were analysed individually. No correlation between seizures and specific voxels was found in either oligodendroglioma grade 2 or grade 3. Conversely, in grade 2 astrocytoma, a correlation was noted between seizures and tumour presence in voxels A1C3S2 (*p* = 0.044) and A2C3S2 (*p* = 0.044). In grade 3 astrocytoma, similar correlations emerged but in two distinct voxels: A3C1S2 (*p* = 0.025) and A3C3S1 (*p* = 0.025).

### 3.3 Tumour Topography and White Matter Infiltration

**FIGURE 4:**
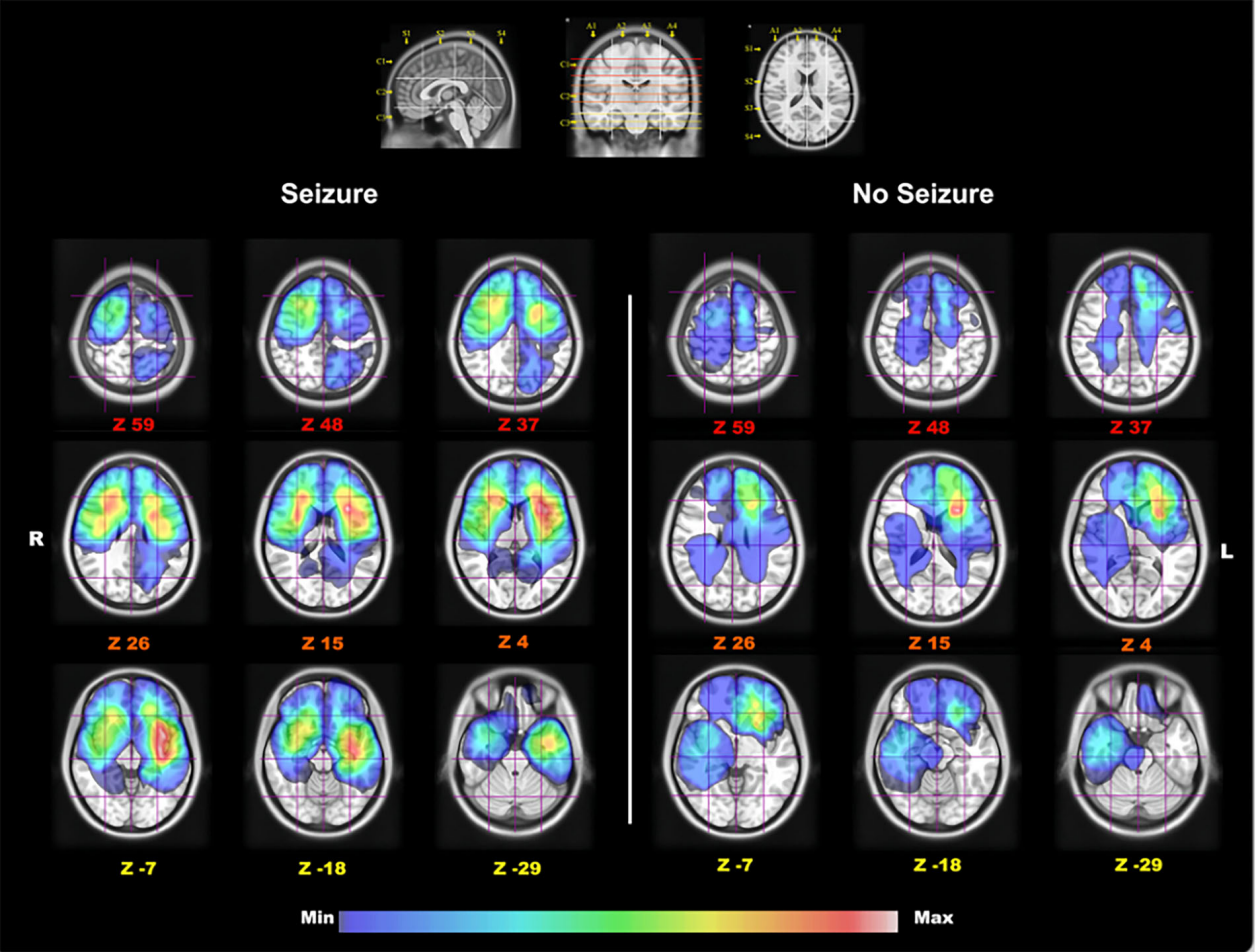
Gradient maps of tumour regions in MNI space, showing Z coordinates for each slice. The upper section includes the Brain‐Grid System with sagittal, coronal, and axial projections as a reference for voxel counts. The lower section shows tumour location frequency for two populations, colour‐graded by voxel infiltration rates (0%‐33%). In the seizure group, tumours are most common in the left fronto‐temporo‐insular region, especially in the insula. In the no‐seizure group, tumours are more prevalent in the deep white matter of t left frontal lobe.

**FIGURE 5:**
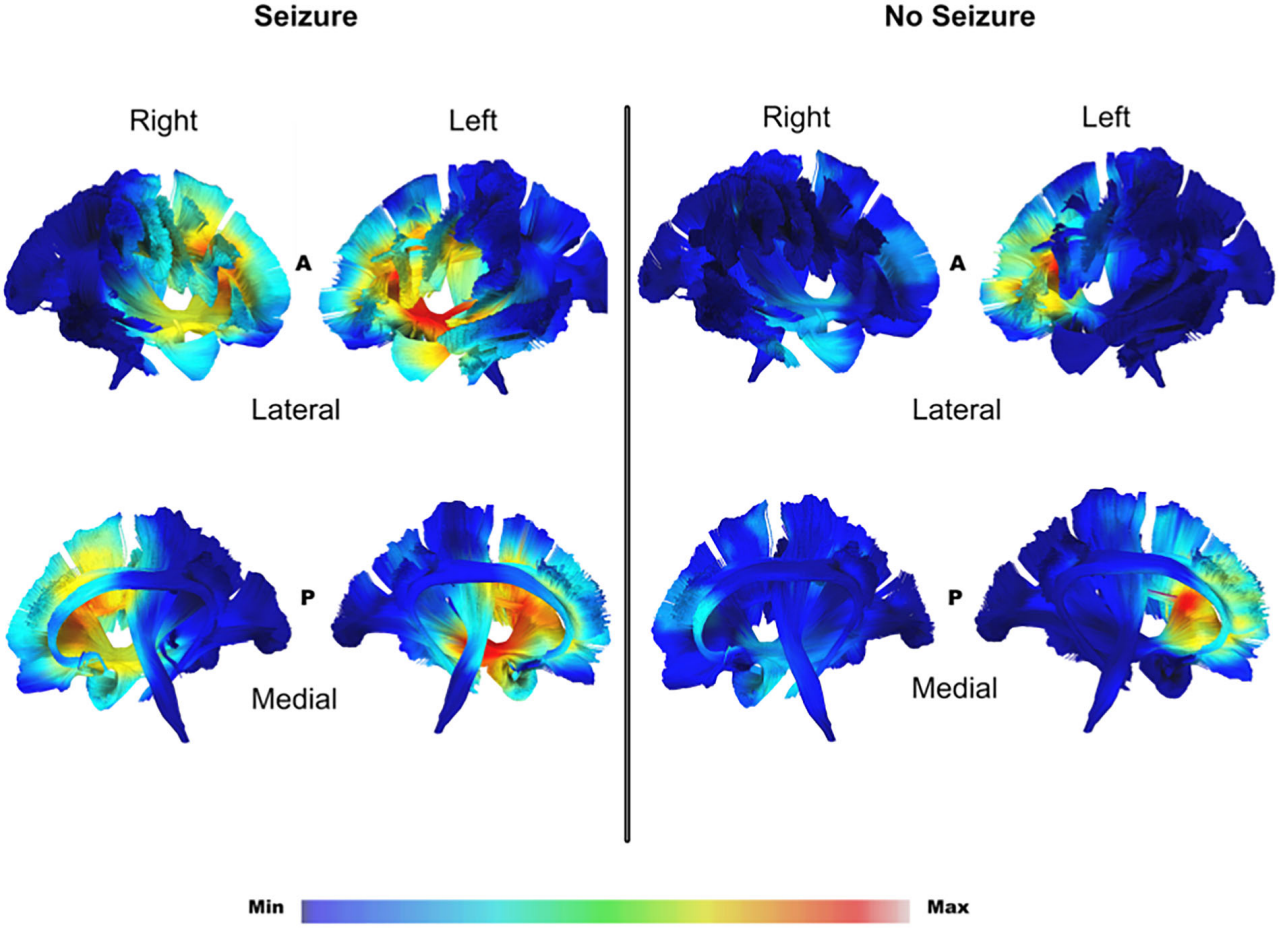
This map highlights differences in white matter involvement between glioma patients with and without preoperative seizures. It combines tumour volumes from 70 patients with seizures and 23 without within the MNI space. Various white matter tracts, including the cingulum, arcuate fasciculus, anterior part of the superior longitudinal fasciculus, inferior fronto‐occipital fasciculus, cortico‐spinal tract, frontal aslant tract, and uncinate fasciculus, were reconstructed and merged with the gradient map for each subgroup, shown in a 3D reconstruction.

The most significant tumour overlap was found in subcortically and differed between the groups, as illustrated in Figures 4 and 5. In the seizure group, tumour occurrence was more extensive and widespread subcortically, particularly in the fronto‐insular regions, while in the no‐seizure group, tumour involvement was more localised to the frontal lobe, particularly the deep white matter.

The colour gradient in Figures 4 and 5, ranging from red to blue, represents tumour occurrence per voxel in each subgroup. In the seizure group, the colour gradient indicates a 16% tumour occurrence, while in the group without seizures, it shows a 33% occurrence. In the seizure group, subcortical involvement is both more extensive and widespread compared to the group without seizures. The IFOF in the seizure group is more subcortical and frontal‐insular in nature, whereas in the no‐seizure group, IFOF involvement is not as subcortical and not spread across different brain lobes.

## 4 Discussion

Our study confirmed three main findings. First, BTRE was highly prevalent in patients with diffuse gliomas, particularly in males. Second, seizures were predominant initial symptom, occurring in 75% of the patients, consistent with previous studies (Pallud et al. 2014; van Breemen et al. 2007). Third, subcortical tumour location showed a topographical correlation with seizure risk. Additionally, the data reaffirmed that seizures are more common in males with diffuse gliomas (Kerkhof and Vecht 2013), but no association with age and seizure was found as opposed to previous studies (Englot et al. 2016; Rossetti and Stupp 2010). Considering the heterogeneity of the cohort with larger groups of grade 2 tumours than grade 3 probably explains the different results.

### 4.1 Tumour Features and Seizure Risk

The associations between voxels A4C2S3 and A3C2S1 and seizure risk identified in the univariate analyses as well as in the multivariate model. This indicates that the observed relationships are independent of potential confounding factors such as age, sex, and tumour volume. These findings suggest that these voxels may play a critical role in the pathophysiology of seizure risk in diffuse gliomas and warrant further investigation. However, the broad confidence interval in A4C2S3 may reduce the reliability of the result. This is likely attributable to the sample size. Despite this, the lower bound being above 1 supports a positive association, warranting further investigation in larger cohorts. In contrast, tumour presence in voxel A3C1S1 was associated with a reduced seizure risk. This suggests that involvement of this region may have a protective effect or be linked to tumour characteristics that are less epileptogenic. Given the relatively narrow confidence interval, this finding appears robust, though the underlying mechanisms remain unclear. It needs to be determined whether structural or functional factors in this region contribute to a lower seizure susceptibility.

In the logistic regression analysis, there was no significant association between the number of voxels affected by the tumour and seizure risk. The odds ratio was close to 1 (OR = 0.994), indicating a negligible effect of voxel count on seizure occurrence. Indeed, the total number of voxels involved does not seem to be a critical determinant of seizure risk.

As mentioned before, controversy exists regarding the correlation between seizure occurrence and tumour location (Englot et al. 2016; Tang et al. 2024; J. Zhang et al. 2019). In our study, when analysing the topography and preferential locations of the two groups (with and without seizures), most tumours were located on the left side in both groups. The main difference was found in insular/subcortical insular voxels in BTRE patients, compared to a more anterior and dorsal distribution within the external capsule region in patients without seizures. Consistent with previous research, our study highlighted the lack of a direct correlation between seizure onset and tumour volume or radiological border suggesting that seizure frequency and onset are not directly dependent on tumour size (Pallud et al. 2014; Smits et al. 2015).

In addition to the odds ratio (OR) of 3.5 for diffuse borders, we calculated the relative risk (RR) to provide a clearer picture of the observed trend. Among patients with sharp borders, 12 out of 54 (22%) experienced seizures, compared to 11 out of 28 (39%) in those with diffuse borders. This corresponds to a relative risk (RR) of 1.78 (39%/22%), suggesting a higher risk of seizures associated with diffuse borders. Although this finding is not significant, it is a highly clinically relevant trend and needs to be further explored.

When comparing tumour types and grades individually the result differed. In all oligodendrogliomas, a reduced seizure risk was shown in A1C3S3. However, when grades 2 and 3 were analysed separately, no correlation with seizures was found. Astrocytoma showed a different pattern, with a significant correlation to seizures both in grades 2 and 3 combined and within each grade separately. Oligodendroglioma are more often cortically distributed and thereby associated with a higher seizure risk compared to astrocytoma that more frequently occur white matter structures (Audrey et al. 2022). Previous studies have shown that tumour occurrence within subcortical structures, bilateral location and deep white matter infiltration are associated with lower seizure risk (Akeret et al. 2019; Kerkhof and Vecht 2013). It has been established that seizure risk is reduced with an increase in tumour grade (Kerkhof and Vecht 2013; Mader et al. 2022; Ollila and Roivainen 2023). Other potential differences in seizure risk between grade 2 and grade 3 tumours, particularly regarding their location and white matter involvement, have not been identified in previous research; further studies are required to verify these findings.

### 4.2 Tumour Topography and White Matter Infiltration

Diffuse glioma with and without seizures differ in the white matter involvement with tumours associated with seizures showing more pronounced changes in the IFOF, SLF, and corona radiata (H. Zhang et al. 2022; S. Zhang et al. 2023). When tumour growth damages white matter, it significantly impacts connectivity and neurological function. While certain compensatory mechanisms can mitigate connectivity loss, excessive compensatory mechanisms may lead to epileptic activity (Szalisznyo et al. 2013). The etiopathogenesis of diffuse glioma is unclear, but it typically appears during adolescence in anterior and associative cerebral areas, which are myelinated later in brain development, suggesting that white matter myelination may play a role in diffuse glioma development. Additionally, white matter fibres and myelin characteristics contribute to tumour diffusion, as glioma cells migrate along subcortical pathways, especially when demyelinated, potentially causing extensive parenchymal invasion and functional disturbances (Duffau 2022).

**FIGURE 6:**
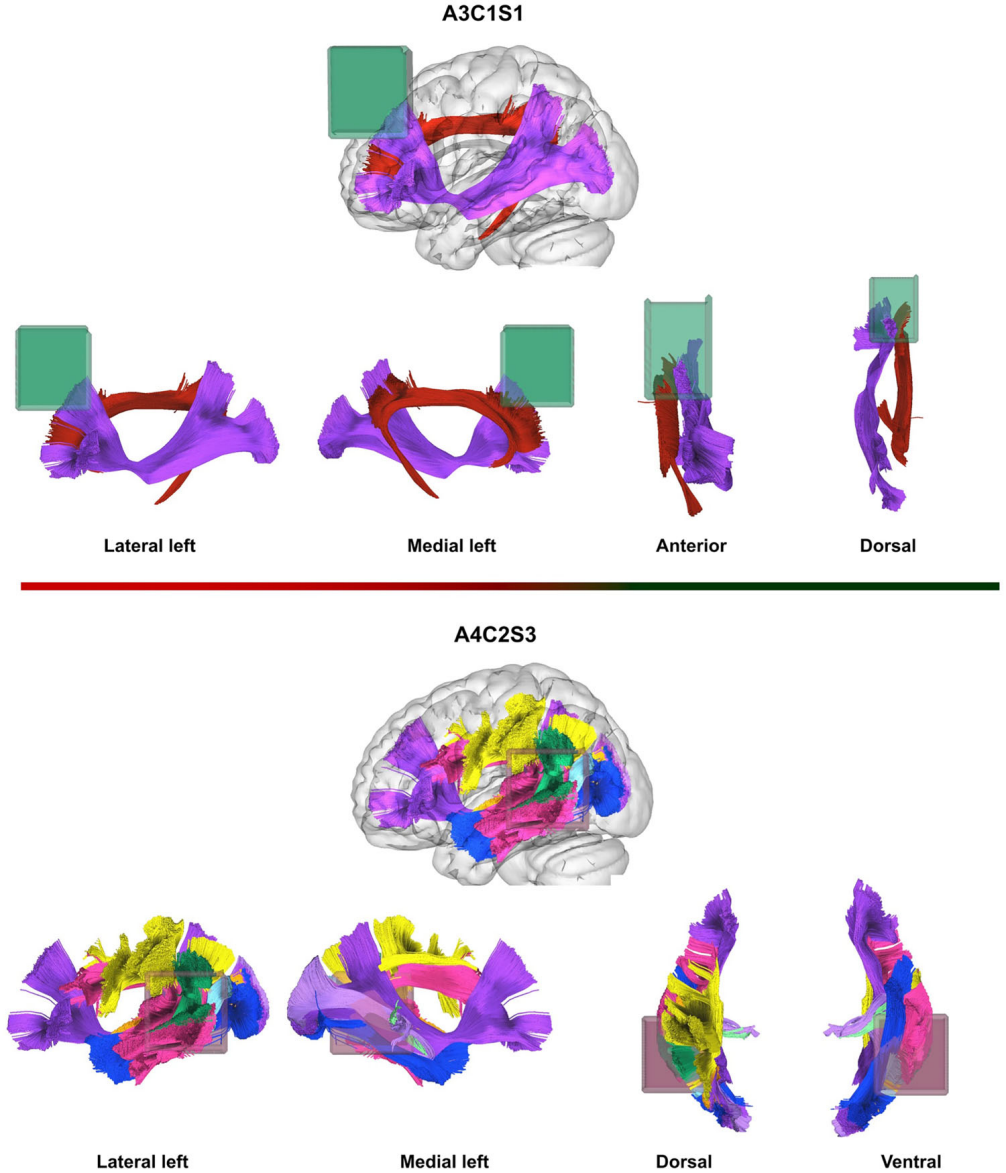
Illustrating the difference in connectivity between the significant voxels. A3C1S1 with involvement of the inferior fronto‐occipital fascicle, IFOF (purple) and cingulum (red). A4C2S3 consisting partly of the IFOF (purple), the anterior indirect component of superior longitudinal fascicle, SLF (yellow), the posterior indirect component of SLF, (green), Arcuate fasciculus, AF (pink) and the inferior longitudinal fascicle, ILF (blue), optic radiation (lavender), acoustic radiation (sea foam green).

In terms of white matter connectivity, the A3C1S1 region contains mostly association pathways, including some frontal terminations of the IFOF and the cingulum. In contrast, the A4C2S3 voxel serves as a junction for several association pathways (including the AF, IFOF, Inferior Longitudinal Fasciculus (ILF), Middle Longitudinal Fasciculus (MdLF), and SLF subsegments), possibly the vertical occipital fascicle (VOF), and projection pathways (including the auditory and optic radiations) (Figure 6).

The preferential location of BTRE in our study also reflected the type and amount of white matter networks involved. In the seizure group, there was predominant involvement of peri‐subinsular connectivity, including the IFOF, UF, and AF on the left side. In the group without seizures, the highest white matter infiltration was found at the level of the dorsal portion of the external capsule, involving only the IFOF. Few studies focus on white matter involvement in BTRE, and hopefully our results may contribute to the understanding of the importance of white matter network as modulators of epileptogenesis (Duffau 2022; Rudà et al. 2012; H. Zhang et al. 2022).

The voxels that highly correlated with seizures was not the same as the hotspot for tumour infiltration, aligning with previous knowledge that peritumoural areas are responsible for seizure onset, rather than the tumour core, in response to frontline tumour cells and the related disconnection of large‐scale networks.

The invasion of a single voxel does not necessarily imply that the voxel is devoid of intrinsic of white matter fibres and completely disconnected. This is illustrated by the two different tumour invaded voxels leads to different consequences in terms of different adaptive reorganisation. Our hypothesis is that the invasion of the A4C2S3 voxel by tumour cells may lead to the disconnection of larger multilobar networks, thereby reducing the mechanisms of cortical adaptation and recruitment for higher‐order functions.

### 4.3 Limitations

The study's retrospective nature and reliance on medical records data introduce several limitations. 276 patients were excluded due to changes in the WHO classification system for CNS tumours over time. Initial treatment guidelines varied, leading to a lack of early histopathological diagnoses. Furthermore, a possible drawback of the BG system is its reliance on fixed anatomical landmarks and the use of atlas‐based MNI space. Possible variations in the normalisation‐registration process may also affect the final count of the infiltrated voxels. Moreover, difference in resolution and acquisition parameters between the cohorts is regarded as a limitation which can bias the segmentation of the tumours. Still, because of the gross delineation of the BG system we don't expect errors in the segmentation to significantly alter the analysis performed. Additionally, the upgrade of the MR scanner and the transition from 2D imaging to 3D imaging was reasonable in terms of gain of image quality and imaging speed. As this was a retrospective study covering several years, a major upgrade to the MR scanner was unavoidable and necessary in a clinical sense to apply. Still, all image datasets were reviewed for the quality and accuracy of registration as previously published and validated by our group. While individual tumour cases may cause variations in anatomy, these do not significantly affect group‐level calculations with gradient maps, where infiltration frequency of each voxel is assessed. Finally, for the white matter analysis, only eight major white matter bundles were included in the gradient maps. This choice was made to better display the relationships between regions and specific white matter bundles. However, several other white matter bundles could be considered to better understand the complexity of subcortical regions.

A disadvantage of the chosen study design, which involves the consecutive inclusion of all patients with oligodendroglioma and astrocytoma, grade 2 and 3, is the resulting heterogeneity of the study population. This diversity, particularly with grade 3 tumours, limits the statistical power due to small sample sizes, making it challenging to detect true effects in the study. As no adjustment for multiple comparisons was performed, the risk of false‐positive findings is acknowledged. This approach was chosen to prioritise the identification of potential voxel‐specific associations with seizure risk.

## 5 Conclusion

This sublobar localisation method aids in understanding the invasive nature of tumours and their correlation with seizure activity. The research establishes a clear association between specific brain regions and the importance of white matter connectivity in relation to risk of seizures. This may provide a basis for future targeted therapeutic algorithms. Future studies with larger cohorts will help to confirm these initial observations and enhance their application in clinical practice.

## Author Contributions

**Ann Westermark**: Conceptualisation, investigation; writing–original draft, methodology, validation, visualisation, writing–review and editing, formal analysis, project administration, software, resources, data curation. **Markus Fahlström**: Methodology, validation, writing–review and editing, visualisation, supervision, resources, formal analysis, software, investigation, writing–original draft. **Sadia Mirza**: Software, formal analysis, visualisation, writing–review and editing. **Maria Zetterling**: Writing–review and editing, methodology, conceptualisation. **Eva Kumlien**: Conceptualisation, investigation, funding acquisition, writing–original draft, methodology, validation, writing–review and editing, supervision, data curation, formal analysis, visualisation. **Francesco Latini**: Supervision, resources, conceptualisation, investigation, writing–original draft, methodology, validation, visualisation, writing–review and editing, software, formal analysis, project administration, data curation.

### Peer Review

The peer review history for this article is available at https://publons.com/publon/10.1002/brb3.70477

## Data Availability

The data that support the findings of this study are available from the corresponding author upon reasonable request.

## 1

Voxels excluded due to lack of tumour growth: A1C1S4, A1C2S4, A1C3S4, A2C2S4, A2C3S4, A3C3S4, A4C3S4.

**TABLE A1.** Univariate logistic regression analysis.

Voxel	OR	*p* Value	95% CI	Lower CI	Upper CI
A1C1S2	2.9	0.19	(0.6–13.6)	0.6	13.6
A1C1S3	1.4	0.71	(0.3–6.9)	0.3	6.9
A1C2S2	1.9	0.26	(0.6–5.7)	0.6	5.7
A1C2S3	1.2	0.76	(0.3–4.9)	0.3	4.9
A1C3S2	0.6	0.35	(0.2–1.8)	0.2	1.8
A2C1S1	1.1	0.90	(0.3–3.7)	0.3	3.7
A2C1S2	0.9	0.86	(0.3–2.7)	0.3	2.7
A2C1S3	0.9	0.86	(0.3–2.8)	0.3	2.8
A2C1S4	0.7	0.51	(0.2–2.2)	0.2	2.2
A2C2S1	0.8	0.74	(0.3–2.6)	0.3	2.6
A2C2S2	0.9	0.86	(0.3–2.7)	0.3	2.7
A2C2S3	1.1	0.88	(0.3–4.4)	0.3	4.4
A2C3S1	1.8	0.49	(0.4–8.6)	0.4	8.6
A2C3S2	0.4	0.08	(0.1–1.1)	0.1	1.1
A3C1S2	0.5	0.17	(0.2–1.4)	0.2	1.4
A3C2S1	0.6	0.35	(0.2–1.7)	0.2	1.7
A3C2S2	0.7	0.51	(0.3–1.9)	0.3	1.9
A3C2S3	3.4	0.13	(0.7–15.9)	0.7	15.9
A3C3S1	0.4	0.16	(0.1–1.4)	0.1	1.4
A3C3S2	1.1	0.91	(0.3–3.3)	0.3	3.3
A4C1S1	0.4	0.25	(0.1–1.7)	0.1	1.7
A4C1S2	1.2	0.70	(0.4–3.8)	0.4	3.8
A4C1S3	.	1.00		.	.
A4C2S1	0.7	0.47	(0.2–1.9)	0.2	1.9
A4C2S2	1.0	0.96	(0.4–2.5)	0.4	2.5
A4C3S2	1.8	0.38	(0.5–7.0)	0.5	7.0
A1C1S1	609113465.337	0.99	0		
A1C2S1	2.864	0.186	(0.603–13.610)	.603	13.610
A1C3S1	580561271.650	0.999	0		
A1C3S3	0.288	0.098	(0.066–1.261)	0.066	1.261
A2C3S3	0.213	0.055	(0.044–1.034)	0.044	1.034
A3C1S3	1.549	0.594	(0.31–7.754)	0.31	7.754
A3C1S4	554565990.829	0.999	0		
A3C2S4	546410608.611	0.999	0		
A3C3S3	2.839	0.338	(0.336–24.007)	0.336	24.007
A4C1S4	538491614.284	1	0		
A4C2S4	538491614.284	1	0		
A4C3S1	0.299	0.158	(0.056–1.596)	0.056	1.596
A4c3s2	1.818	0.382	(0.476–6.951)	0.476	6.951
